# Growing Interface with Phase Separation and Spontaneous Convection during Hydrodynamically Stable Displacement

**DOI:** 10.3390/ma14206089

**Published:** 2021-10-14

**Authors:** Takahiko Ban, Ryohei Tanaka, Ryuta X. Suzuki, Yuichiro Nagatsu

**Affiliations:** 1 Division of Chemical Engineering, Department of Materials Engineering Science, Graduate School of Engineering Science, Osaka University, Machikaneyamacho 1-3, Toyonaka, Osaka 560-8531, Japan; 2 Department of Chemical Engineering, Tokyo University of Agriculture and Technology, Nakacho 2-24-16, Koganei, Tokyo 184-8588, Japan; ryohei05171994@gmail.com (R.T.); ryuta.x.suzuki@gmail.com (R.X.S.); nagatsu@cc.tuat.ac.jp (Y.N.)

**Keywords:** fluid displacement, Family–Vicsek scaling, phase separation

## Abstract

The displacement of one fluid by another is an important process, not only in industrial and environmental fields, such as chromatography, enhanced oil recovery, and CO_2_ sequestration, but also material processing, such as Lost Foam Casting. Even during hydrodynamically stable fluid displacement where a more viscous fluid displaces a less viscous fluid in porous media or in Hele-Shaw cells, the growing interface fluctuates slightly. This fluctuation is attributed to thermodynamic conditions, which can be categorized as the following systems: fully miscible, partially miscible, and immiscible. The dynamics of these three systems differ significantly. Here, we analyze interfacial fluctuations under the three systems using Family–Vicsek scaling and calculate the scaling indexes. We discovered that the roughness exponent, α, and growth exponent, β, of the partially miscible case are larger than those of the immiscible and fully miscible cases due to the effects of the Korteweg convection as induced during phase separation. Moreover, it is confirmed that fluctuations in all systems with steady values of α and β are represented as a single curve, which implies that accurate predictions for the growing interface with fluctuations in Hele-Shaw flows can be accomplished at any scale and time, regardless of the miscibility conditions.

## 1. Introduction

When a more viscous fluid displaces a less viscous fluid in porous media or in a Hele-Shaw cell, which is comprised of two parallel plates with a thin gap between them, the interface between the two fluids becomes stable. However, in the reverse situation, where a less viscous fluid displaces a more viscous fluid, the interface becomes hydrodynamically unstable and forms finger-like patterns. This phenomenon is known as the Saffman–Taylor instability [1] or viscous fingering [2,3]. Hydrodynamic instabilities in the Hele-Shaw cell have been utilized in a step-emulsification process for the high-throughput production of colloidal monodisperse droplets [4] and the flow-driven control of calcium carbonate precipitation patterns [5]. Especially in so-called Lost Foam Casting, it has been pointed out that deformation of the liquid metal interface due to Saffman–Taylor instability leads to the entrainment of the degrading pattern material and associated defects [6]. Therefore, establishing of a quantitative prediction of the deformation due to such hydrodynamic instability is important for material design. The fluid pair is thermodynamically categorized into the following three types. The first is a fully miscible system in which the fluids have infinite mutual solubility, such as glycerol–water. The second is a partially miscible system where fluids have finite mutual solubility, such as crude oil–water under high pressure and temperature conditions for enhanced oil recovery [7,8]. The third is an immiscible system having zero mutual solubility of fluids, such as oil–water at atmospheric pressure and room temperature (25 ± 1 °C). Several studies regarding interfacial hydrodynamics that considered fully miscible and immiscible cases have been reported [3,9,10,11,12]. Recently, Suzuki et al. discovered a novel interfacial dynamics feature using a partially miscible system, which can be treated at room temperature and atmospheric pressures [13,14,15,16]. Their partially miscible system was comprised of polyethylene glycol (PEG), Na_2_SO_4_, and water, for which spinodal decomposition phase separation occurred at the boundary of the displacing and displaced liquids. They performed hydrodynamically stable displacements in a radial Hele-Shaw cell and observed interfacial deformation only in the partially miscible system [13]. They proved that interfacial fluctuations are driven by spontaneous convection as induced by the Korteweg force [17,18] due to the chemical potential gradient during spinodal decomposition-type phase separation. The Korteweg force was first proposed by Korteweg in 1901 [19]. It is defined thermodynamically as the functional derivative of free energy [20], and is characterized as a body force. The Korteweg force tends to minimize the free energy stored at the interface and induces spontaneous convection. Suzuki et al. found that the direction of Korteweg convection differed from that of injection, which roughened the interface [13].

Interfacial fluctuations are ubiquitous in nature at various lengths and time scales. They include the propagation of flame fronts, as well as deposition processes, such as falling snow, atomic deposition, and bacterial growth [21]. In general, the interface morphology depends on the length and time scale of observations. In such growing interfaces, it has been reported that a universal scaling law holds theoretically [21,22]. Interface fluctuations are often quantified by the width w(l,t), which is defined as the standard deviation of the interface height h(x,t) over a length scale l at time t. Family–Vicsek scaling is one of the most popular scaling laws [23] as it expresses the self-affinity of an interface as:

(1) $$w\left(l,t\right)\sim t^{\beta }F\left(lt^{-\frac{1}{z}}\right)\sim \{\begin{array}{c} l^{\alpha }\;\mathrm{for}\;l\ll l_{*}\\ t^{\beta }\;\mathrm{for}\;l\gg l_{*} \end{array},\;$$

where α and β are the exponents that characterize the length- and time-dependent dynamics of the roughening process, respectively; z=α/β is a dynamic exponent; F(·) is a scaling function; and $l_{*}\sim t^{1/z}$ is the crossover length scale. The simplest theory for a local growing interface was proposed by Kardar, Parisi, and Zhang and is known as the KPZ theory [22]. In two dimensions, the renormalization group approach provides the exact value of the exponents at $\alpha^{\mathrm{KPZ}}$ = 1/2 and $\beta^{\mathrm{KPZ}}$ = 1/3, which are universal as widely confirmed in numerical models [21]. An experimental example regarding the propagating fronts of combustion was reported by Myllys et al. [24]. They discovered that 0.51 ≤α≤ 0.57 ($\chi_{LR}$ in their study) and 0.28 ≤β≤ 0.40 ($\beta_{LR}$ in their study). Another study pertaining to the interface growth of bacterial colonies was reported by Wakita et al. [25], who observed that α ≅ 0.50 ± 0.01 for one condition and 0.76 ≤α≤ 0.80 for another. Takeuchi et al. [26] demonstrated that α=0.50 and β = 0.336 at the growing interface in nematic liquid crystals.

The roughness of the growth interface for various phenomena was analyzed via scaling. Fluid displacement occurs frequently in industrial and environmental improvements [8,27,28]; however, it progresses at various lengths and time scales. In actual industrial processes, fluid displacement not only involves hydrodynamic instabilities but also various irreversible processes, such as mutual diffusion, phase separation associated with composition changes, and forced convection due to external forces. Studies regarding interfacial fluctuations that involve such complex irreversible processes have not been reported to date because a solution system that controls its solubility under normal temperatures and pressures with nearly unchanged physicochemical properties has not yet been discovered. We found a solution system in a previous study that can significantly change its thermodynamic state with only small changes in composition at room temperature and atmospheric pressures while maintaining its component viscosities [13]. If the interfacial fluctuations of fluid displacements in various nonequilibrium conditions observed at laboratory scales follow the scaling law, then it can be applied to various industrial processes that are performed on large scales. In this study, the Family–Vicsek scaling is used to quantitatively evaluate the roughness of the growth interfaces in hydrodynamically stable fluid displacements using three systems with the following thermodynamic states: fluid displacement with mutual diffusion (fully miscible system), without mutual diffusion (immiscible system), and with phase separation (partially miscible system). We discovered that the characteristic exponents of the roughening process depend on its thermodynamic state.

## 2. Materials and Methods

### 2.1. Solutions

**Figure 1:**
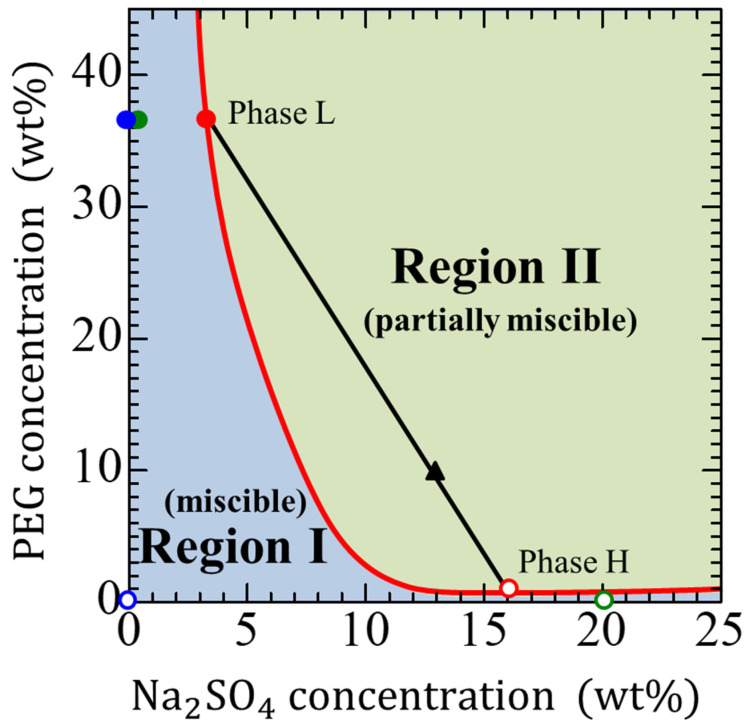
Phase diagram of the polyethylene glycol (PEG)–Na_2_SO_4_–water system [15]. The different color symbols show the three systems having different three thermodynamic states: the red circles for the immiscible system, the blue circles for the fully miscible system, and the green circles for the partially miscible system. The closed circles mean more viscous solutions and open ones mean less viscous solutions. The black line is the tie-line, whose end points determine the equilibrium phase compositions. The red curve represents the binodal curve, indicating equilibrium compositions of the two immiscible phases after separation.

We used the same solutions indicated in previous studies, [13,14] i.e., a 36.5 wt% PEG–0 wt% Na_2_SO_4_ solution system as fully miscible, a 36.5 wt% PEG–20 wt% Na_2_SO_4_ solution system as partially miscible, and a phase L–phase H (explained below) as immiscible (Figure 1), where PEG represents polyethylene glycol 8000, whose average molecular weight is approximately 8000. Figure 1 presents the phase diagram [29] of PEG–Na_2_SO_4_–water at 25 °C. When the composition of the solution is in Region I, the solution approaches a single phase, which is known as a fully miscible system. The displacement of a fully miscible system is thermodynamically stable, but the molecular diffusion progresses at the interface and the fluids mix together. When the composition is in Region II, the solution separates into two phases. The red solid curve in the figure shows the immiscible composition. Thus, when the initial solution compositions are on the curve, they are immiscible. For example, when the solution is comprised of 10 wt% PEG and 13 wt% Na_2_SO_4_ (closed black triangle in Figure 1), it separates into phases L (closed red circle) and H (open red circle). Phase L is comprised of 36.5 wt% PEG and 3.2 wt% Na_2_SO_4_, and phase H is comprised of 1.4 wt% PEG and 16.0 wt% Na_2_SO_4_. Phases L and H are in thermodynamic equilibrium with each other. and considered immiscible. The displacement between the two fluids with a composition within Region II becomes thermodynamically unstable, and the mixing of the two fluids causes phase separation to occur at the interface. Therefore, the composition of a system is important to determine the thermodynamic state of a solution. Using the phase diagram in Figure 1 allows for the easy controlling the thermodynamic state of the solution system. The physical properties used in the system, such as the density and viscosity, are listed in Table 1.

### 2.2. Displacement

**Figure 2:**
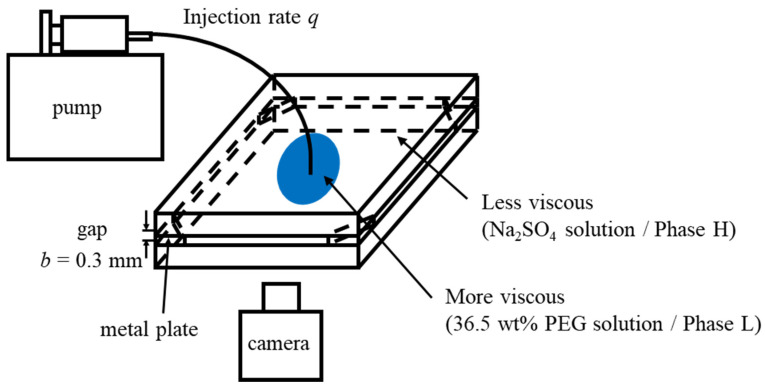
Schematic illustration of fluid displacement experiments [15].

We performed fluid displacement under hydrodynamically stable conditions, i.e., a more viscous fluid displaces a less viscous fluid. Therefore, a 36.5 wt% PEG solution was used to displace a 0 wt% Na_2_SO_4_ solution for the fully miscible case; a 36.5 wt% PEG solution to displace a 20 wt% Na_2_SO_4_ solution for the partially miscible case; and phase L to displace phase H for the immiscible case (shown in Table 1). A radial Hele-Shaw cell comprises two square transparent glass plates (140 mm × 140 mm × 10 mm) with a gap of 0.3 mm. The gap was achieved using four metal plates located in four corners of the cell. The top glass plate had a small hole (4 mm diameter) drilled into the center for fluid injection. We first filled the cell with the less viscous liquid, then injected the more viscous liquid into it, as shown in Figure 2. The displacement experiments were recorded from the bottom of the setup using a video camera. To visualize the displacement, the more viscous fluids (PEG solution and phase L) were dyed blue with 0.1 wt% indigo carmine, which could not dissolve into the Na_2_SO_4_ solution due to the salting-out effect. All experiments were performed at room temperature (25 ± 1 °C) and atmospheric pressures at a constant injection flow rate of $q=7.07\times 10^{-10}{\;m}^{3}/s.$ We conducted the fluid displacement under same conditions three times to confirm the reproductivity.

### 2.3. Family–Vicsek Scaling

**Figure 3:**
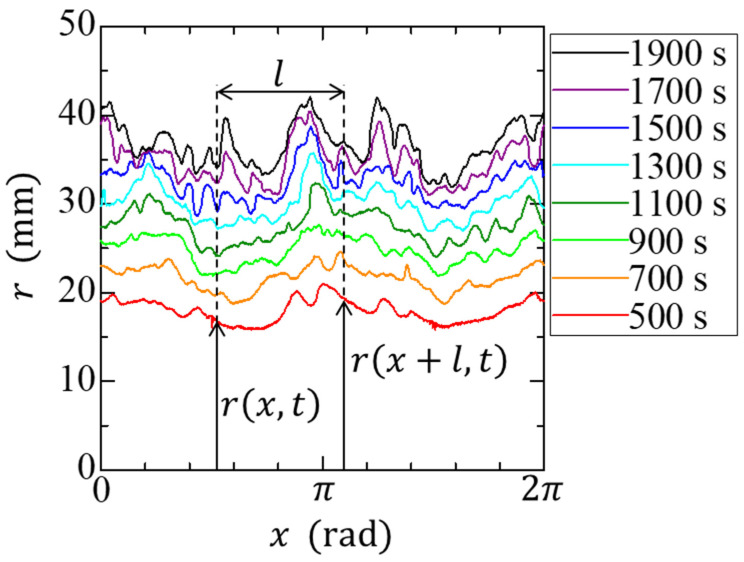
Example of the time evolution of the interface at t = 500–1900 s. The value l is determined by r and x and has the unit of rad.

We define the local radius r(x,t) for the growing interface to characterize its roughness as a function of the coordinate x. The radius of the growth interface at each time is expressed as a function of angle (Figure 3). The interface width is defined as follows [21]:

(2) $$w\left(l,t\right)\equiv \langle {\sqrt{\langle {\left[r\left(x,t\right)-{\langle r\rangle }_{l}\right]}^{2}\rangle }}_{l}\rangle ,\;$$

where l is a length segment along the interface, 〈 〉${}_{l}$ denotes the average over a length segment l, and 〈 〉 denotes the average of the entire interface. Therefore, w(l,t) is regarded as a root-mean-squared interface width at time t. The temporal growth of the roughness was measured based on the overall width as:

(3) $$W\left(t\right)\equiv \sqrt{\langle {\left[r\left(x,t\right)-\langle r\rangle \right]}^{2}\rangle }\;.\;$$

We then calculated the roughness exponent α and growth exponent β for the three miscibility systems using Equations (1)–(3).

## 3. Results and Discussion

**Figure 4:**
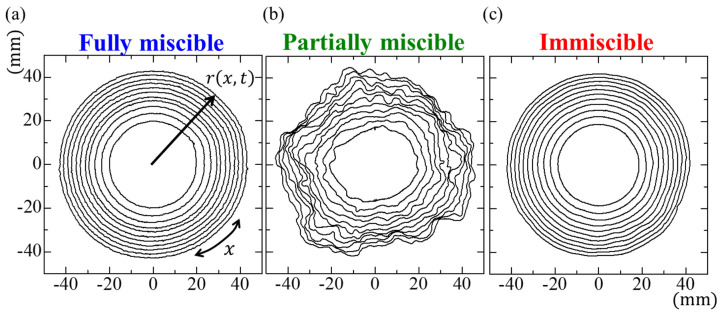
Time evolution of the interface for the (**a**) fully miscible, (**b**) partially miscible, and (**c**) immiscible systems. The result lines are shown every 200 s ranging from *t* = 500−2500 s.

Figure 4 presents a typical example of the growing interface during fluid displacement for the three miscibility systems. A nearly circular pattern was observed in the fully miscible and immiscible systems, albeit with slight distortions because the experimental conditions (a more viscous fluid displaced a less viscous fluid in the horizontal Hele-Shaw cells) were hydrodynamically stable according to Darcy’s law for Hele-Shaw flows [1]. In a fully miscible system, the interface diffuses due to the mutual dissolution of both phases. Therefore, the contour of the binarized interface of the fully miscible system appears to grow faster than that of the immiscible system. However, for the partially miscible system, distinct distortions appeared at the growing interface due to spontaneous convection by the Korteweg force, which was induced by compositional gradients during phase separation [13,14]. The interface distortions increased over time because the flow weakened due to the radial geometry, despite a constant volume from the injection flow rate.

**Figure 5:**
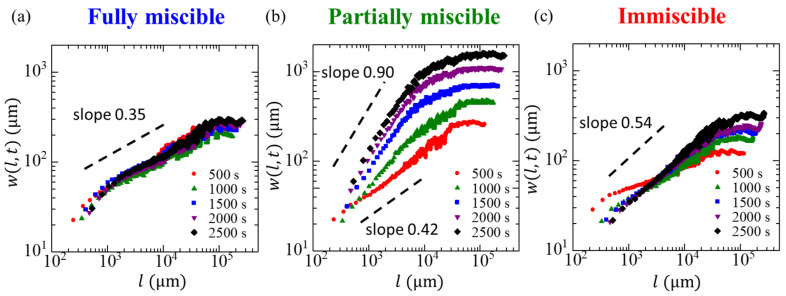
Scaling of the width w(l,t) at different times t for the (**a**) fully miscible, (**b**) partially miscible, and (**c**) immiscible systems. The various colors indicate different times, and the dashed lines serve as visual guides.

Figure 5 presents the scaling of the interface width w(l,t) with the length scale l for different measurement times. In the present study, the onset time of the injection is defined as t=0 and x and l are measured in length scale from radii. For all systems, the interface width increased with the length scale and reached a steady state when beyond a certain length, which increased with the measurement time. The dependence of the interfacial width on the length scale in the partially miscible system was more significant than that of the other two systems, and a considerable temporal change was observed. The slope in the fully miscible case differs slightly from that in the immiscible case because of diffusion in the fully miscible case and interfacial tension in the immiscible case. The slope of the data shown in Figure 5 represents the roughness exponent α. The used value of α is the value when the α achieved at steady-state value.

**Figure 6:**
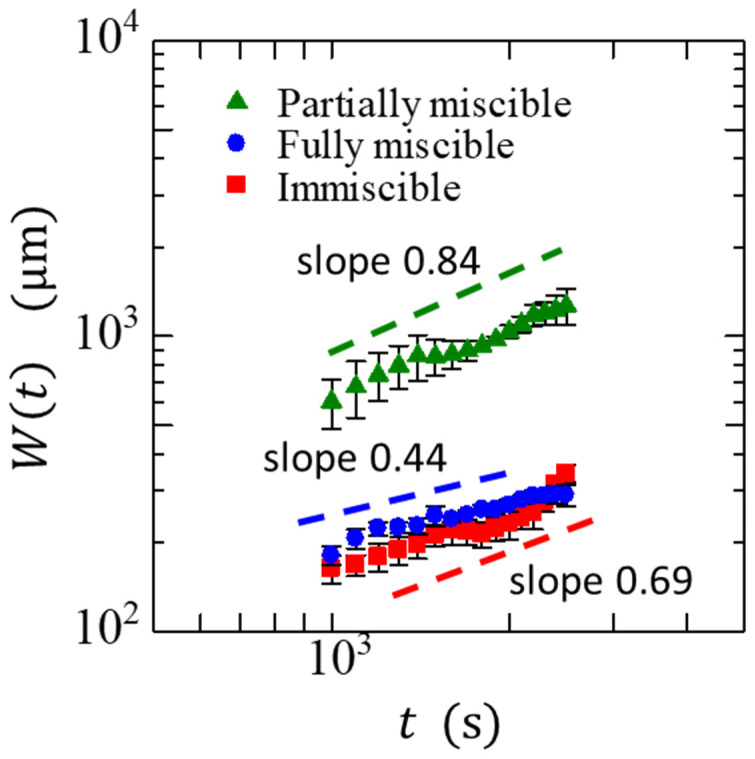
Time evolution of the overall width W(t). The various colors indicate different miscibilities, and the dashed lines serve as visual guides. The slope is calculated at the region where the value of α. is constant.

Figure 6 presents the time dependence of the overall width W(t). After the initial disturbance, W(t) began to increase over time. The β remains nearly constant in the region where α reaches a steady state with respect to time. We obtained β for the three different systems as 0.84 ± 0.17, 0.69 ± 0.05, and 0.44 ± 0.12 for the partially miscible, immiscible, and fully miscible systems, respectively. The α and β in the partially miscible system were significantly higher than those predicted by various models without spatially nonlocal interactions [22,23,24]. Such high values of the exponents in the partially miscible system were most likely because the phase separation affected the fluctuations of the interfacial growth. The roughening of the moving interface based on the phase field model using the generalized Cahn–Hilliard equation, which describes the dynamics of composition change with phase separation, yielded β = 0.85 ± 0.04 [30,31]. In this model, α varied significantly from 0.34 to 1.4 with a decreasing disorder strength [32]. When phase separation occurred and nonlocal interactions became dominant through the effects of interfacial tension and liquid conservation, both exponents increased. As the effect of fluctuation became dominant, α approached the value predicted by the models without spatially nonlocal interactions. The immiscible interface is affected more by fluctuation than the partially miscible interface. Our experimental results show that α. for the immiscible interface is lower than that for the partially miscible interface, which is consistent with the model predictions.

**Figure 7:**
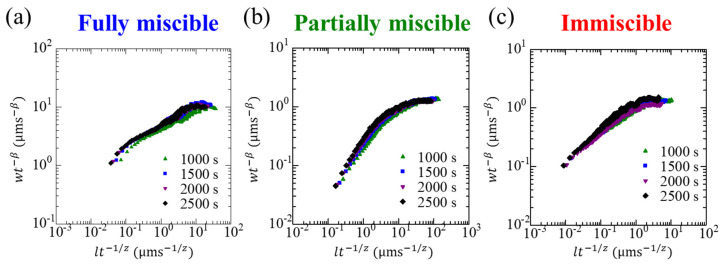
Collapse of data using Equation (1) for the (**a**) fully miscible, (**b**) partially miscible, and (**c**) immiscible systems presenting Family−Vicsek scaling.

We demonstrated the correlation of the interface width using Family−Vicsek scaling with α. and β obtained in the long-time scale portion (shown in Table 2). We confirmed that our data for w(l,t) collapsed onto a single curve in each system (Figure 7). This indicates that predictions for the fluctuations of the growing interface can be achieved on any spatiotemporal scale, regardless of the thermodynamic state of the solution.

## 4. Conclusions

We demonstrated interfacial fluctuation differences between three thermodynamic conditions (fully miscible, partially miscible, and immiscible systems) in a hydrodynamically stable displacement of liquids using Family–Vicsek scaling. We confirmed that the fluctuations in all systems with steady values of α and β can be represented with a single curve. This implies that accurate predictions for the growing interface with fluctuations in Hele-Shaw flows can be accomplished at any scale and time for applications, regardless of system thermodynamics. The interfacial roughness, α, and interfacial growth, β, of partially miscible systems were larger than those of immiscible and fully miscible systems because of the Korteweg-induced convection during phase separation in the partially miscible systems, which affected the interfacial roughness and growth. The phenomenon in which the roughness exponents are larger than the KPZ predictions is known as anomalous roughening, which is frequently observed in growing interfaces accompanied by fluid flow [33,34,35]. However, this is still an unsolved problem. Moreover, the dependence of the exponential values α and β on the flow rate, viscosity ratio, and concentration is of interest and should be addressed in future studies.

## Figures and Tables

**Table 1:** Physicochemical properties of the considered system.

System	Displacing More Viscous Liquid (Density, Viscosity)	Displaced Less Viscous Liquid (Density, Viscosity)
Fully miscible	36.5 wt% PEG solution (1.07 g/cm^3^, 112 mPa·s)	0 wt% Na_2_SO_4_ solution (0.997 g/cm^3^, 0.972 mPa·s)
Partially miscible	36.5 wt% PEG solution (1.07 g/cm^3^, 112 mPa·s)	20 wt% Na_2_SO_4_ solution (1.19 g/cm^3^, 2.08 mPa·s)
Immiscible	Phase L (1.08 g/cm^3^, 125 mPa·s)	Phase H (1.16 g/cm^3^, 1.76 mPa·s)

**Table 2:** Summary of steady values of α and β for the three experiments.

	Fully Miscible	Partially Miscible	Immiscible
α	0.35 ± 0.02	0.86 ± 0.03	0.49 ± 0.02
β	0.44 ± 0.12	0.84 ± 0.17	0.69 ± 0.05

## Data Availability

The data that supports the findings of this study are available within the article.
